# Supplementary material for: Management of Patients with Hypersensitivity to Platinum Salts and Taxane in Gynecological Cancers: A Cross-Sectional Study by the European Network of Young Gynaecologic Oncologists (ENYGO)
Source: Cancers (Basel). 2024 Mar 14;16(6):1155. doi: 10.3390/cancers16061155 (PMC10969349; doi:10.3390/cancers16061155)
Supplement: Supplementary file 1 [file cancers-16-01155-s001.zip › cancers-2900093-supplementary.pdf]

Survey on the management of patients with hypersensitivity to platinum salts and taxane

Dear Colleagues and friends,

The development of multidisciplinary guidelines, consensus statements and good practice recommendations to enhance cancer management is crucial. Standardising the quality of care is important for best patient care.

In this survey, we want to determine the current management of hypersensitivity reactions (HSR) of platinum salts and taxane chemotherapy at the clinics in Europe.

A major problem occurring with repeated exposure to chemotherapy with platinum salts and taxane based chemotherapy consists of immediate type HSR. About 5% of the general oncologic population is affected by platinum hypersensitivity and 10% by hypersensitivity to taxanes. Identifying patients at risk of HSR is important to ensure patient safety during the chemotherapy. Desensitization should be considered in patients with HSR to platinum salts and taxanes, as it is a safe alternative to continue the standard chemotherapy. However, usually a shift to another substance category occurs corresponding to a next line treatment option.

This questionnaire contains several questions regarding the management of HSR of platinum salts and taxane chemotherapy at your clinic.

Completing the survey should not take more than 7 minutes.

Thank you for your kind help,

Sincerely

ENYGO scientific committee

* 1. What is your gender?

- ○ Female
- ○ Male
- ○ Other (please specify)

* 2. What is your current age (in years)?

* 3. In what country do you currently work?

* 4. In what type of hospital do you currently work?

- [ ] University Hospital
- [ ] Regional Hospital
- [ ] Public Hospital
- [ ] Private Hospital
- [ ] Private practice
- [ ] Other (please specify)

* 5. What is your specialty?

- ( ) Gynaecologic oncologist
- ( ) Gynaecologist
- ( ) Medical oncologist
- ( ) Radiation oncologist
- ( ) Specialist of internal medicine
- ( ) Other (please specify)

* 6. In which clinic do you currently work? (This question and your answer is only for internal use to prevent a selection bias)

* 7. What type of gynaecological cancer do you treat?

- [ ] Ovarian
- [ ] Cervical
- [ ] Vulvar
- [ ] Vaginal
- [ ] Corpus
- [ ] Breast
- [ ] No
- [ ] Other (please specify)

* 8. How many years have you had clinical practice in gynaecologic oncology?

- ( ) Less than 5 years
- ( ) 5-10 years
- ( ) More than 10 years

* 9. Are you involved in chemotherapy treatment of gynaecological cancer patients?

- [ ] Yes
- [ ] No

Survey on the management of patients with hypersensitivity to platinum salts and taxane

Part I - Platinum Hypersensitivity Questions

* 10. How many patients with gynaecological cancer do you treat with platinum salts chemotherapy at your department per year?

- ( ) >300
- ( ) 200-300
- ( ) 100-200
- ( ) 50-100
- ( ) 30-50
- ( ) 20-30
- ( ) 10-20
- ( ) <10
- ( ) Other (please specify)

* 11. How many hypersensitivity reactions to platinum salts do you experience at your department per year?

- ( ) >50
- ( ) 30-50
- ( ) 20-30
- ( ) 10-20
- ( ) 5-10
- ( ) <5
- ( ) Other (please specify)

* 12. What measures are you taking if a hypersensitivity reaction to platinum salts CTC (Common Toxicity Criteria) Grade 1-2 occurs?

- [ ] Premedication with antihistamines/steroids and new attempt with standard infusion
- [ ] Suspension of the chemotherapy
- [ ] Change the chemotherapy to e.g. Oxaliplatin
- [ ] Tolerance induction (stepwise increase of infusion rate of highly diluted platinum dilution)
- [ ] Other (please specify)

* 13. What measures are you taking if a hypersensitivity reaction to platinum salts CTC (Common Toxicity Criteria) Grade 3-4 occurs?

- [ ] Premedication with antihistamines/steroids and new attempt with standard infusion
- [ ] Suspension of the chemotherapy
- [ ] Change the chemotherapy to e.g. Oxaliplatin
- [ ] Tolerance induction (stepwise increase of infusion rate of highly diluted platinum dilution)
- [ ] Other (please specify)

* 14. Do you perform tolerance induction of platinum salts?

- ( ) Yes, at our clinic
- ( ) No, but I refer the patient to another clinic
- ( ) No
- ( ) Other (please specify)

15. If yes, who is performing the tolerance induction of platinum salts?

- [ ] Allergologist
- [ ] Medical oncologist
- [ ] Specialist for internal medicine
- [ ] Gynaecologic oncologist
- [ ] Other (please specify)

16. If no, to which type of clinic are you referring the patients?

- ( ) University Hospital
- ( ) Regional Hospital
- ( ) Public Hospital
- ( ) Private Hospital
- ( ) Private practice
- ( ) Other (please specify)

* 17. How many times can you continue the chemotherapy after tolerance induction of platinum salts?

- every time
- >50%
- <50%
- never
- Other (please specify)

* 18. Did you experience a critical incident event in the course of the tolerance induction of platinum salts?

- Yes, more than once
- Yes, once
- No
- Other (please specify)

19. If yes, what was the reason?

- Hypersensitivity reaction to platinum salts CTC (Common Toxicity Criteria) Grade 1-2
- Hypersensitivity reaction to platinum salts CTC (Common Toxicity Criteria) Grade 3-4
- Death
- Patient not informed about the risks of tolerance induction
- Other (please specify)

* 20. Have you been aware about the possibility of desensitization of patients with HSR to platinum and taxane prior to this survey?

- [ ] Yes
- [ ] No
- [ ] Other (please specify)

* 21. Do you think there is a need to standardise the management of platinum and taxane hypersensitivity reactions in gynaecological cancer and to develop international guidelines?

- ( ) Yes
- ( ) No
- ( ) Other (please specify)

ENYGO

ESGO European Network of Young Gynae Oncologists

Survey on the management of patients with hypersensitivity to platinum salts and taxane

Part II - Taxane Hypersensitivity Questions

* 22. How many patients with gynaecological cancer do you treat with taxane chemotherapy at your department per year?

- ◯ >300
- ◯ 200-300
- ◯ 100-200
- ◯ 50-100
- ◯ 30-50
- ◯ 20-30
- ◯ 10-20
- ◯ <10
- ◯ Other (please specify)

* 23. How many hypersensitivity reactions to taxane do you experience at your department per year?

- ◯ >50
- ◯ 30-50
- ◯ 20-30
- ◯ 10-20
- ◯ 5-10
- ◯ <5
- ◯ Other (please specify)

* 24. What measures are you taking if a hypersensitivity reaction to taxane CTC (Common Toxicity Criteria) Grade 1-2 occurs?

- ☐ Premedication with antihistamines/steroids and new attempt with standard infusion
- ☐ Suspension of the chemotherapy
- ☐ Change the chemotherapy to e.g. Nab-Paclitaxel
- ☐ Tolerance induction (stepwise increase of infusion rate of highly diluted taxane dilution)
- ☐ Other (please specify)

* 25. What measures are you taking if a hypersensitivity reaction to taxane CTC (Common Toxicity Criteria) Grade 3-4 occurs?

- [ ] Premedication with antihistamines/steroids and new attempt with standard infusion
- [ ] Suspension of the chemotherapy
- [ ] Change the chemotherapy to e.g. Nab-Paclitaxel
- [ ] Tolerance induction (stepwise increase of infusion rate of highly diluted taxane dilution)
- [ ] Other (please specify)

* 26. Do you perform tolerance induction of taxane?

- ( ) Yes, at our clinic
- ( ) No, but I refer the patient to another clinic
- ( ) No
- ( ) Other (please specify)

27. If yes, who is performing the tolerance induction of taxane?

- [ ] Allergologist
- [ ] Medical oncologist
- [ ] Specialist for internal medicine
- [ ] Gynaecologic oncologist
- [ ] Other (please specify)

28. If no, to which type of clinic are you referring the patients?

- ( ) University Hospital
- ( ) Regional Hospital
- ( ) Public Hospital
- ( ) Private Hospital
- ( ) Private practice
- ( ) Other (please specify)

* 29. How many times can you continue the chemotherapy after tolerance induction of taxane?

- every time
- >50%
- <50%
- never
- Other (please specify)

* 30. Did you experience a critical incident event in the course of the tolerance induction of taxane?

- Yes, more than once
- Yes, once
- No
- Other (please specify)

31. If yes, what was the reason?

- Hypersensitivity reaction to platinum salts CTC (Common Toxicity Criteria) Grade 1-2
- Hypersensitivity reaction to platinum salts CTC (Common Toxicity Criteria) Grade 3-4
- Death
- Patient not informed about risks of tolerance induction
- Other (please specify)

ENYGO
ESGO | European Network of Young Gynae Oncologists

Survey on the management of patients with hypersensitivity to platinum salts and taxane

Part III - Outlook

* 32. Have you been aware about the possibility of desensitization of patients with HSR to platinum and taxane prior to this survey?

☐ Yes

☐ No

☐ Other (please specify)

* 33. Do you think there is a need to standardise the management of platinum and taxane hypersensitivity reactions in gynaecological cancer and to develop international guidelines?

○ Yes

○ No

○ Other (please specify)
